# Laparoscopic ileocecal resection can be applied for appendiceal cancer with an ileal fistula: A case report

**DOI:** 10.1016/j.ijscr.2018.10.004

**Published:** 2018-10-08

**Authors:** Junko Mukohyama, Yasuo Sumi, Kiyonori Kanemitsu, Hiroshi Hasegawa, Masashi Yamamoto, Shingo Kanaji, Yoshiko Matsuda, Kimihiro Yamashita, Takeru Matsuda, Taro Oshikiri, Tetsu Nakamura, Satoshi Suzuki, Yoshihiro Kakeji

**Affiliations:** a Division of Gastrointestinal Surgery, Kobe University Graduate School of Medicine, Kobe, Hyogo 6500017, Japan; b Division of Gastrointestinal Surgery, Asahikawa Medical University, Asahikawa, Hokkaido 0788510, Japan; c Dept. of Surgery, Yodogawa Christian Hospital, Osaka, Osaka 5330024, Japan

**Keywords:** Appendiceal cancer, Fistula, Laparoscopic surgery, Case report

## Abstract

**Introduction:**

Primary appendiceal cancer with fistula formation is extremely rare. We report a case of a patient with appendiceal cancer invading the ileum who underwent successful laparoscopic ileocecal resection.

**Presentation of case:**

A 76-year-old man who presented with fever and abdominal pain was diagnosed with acute appendicitis and received antibiotics at a local hospital. After a few days, he was referred to our hospital because of an abnormality found in the colonoscopy, which was an oozing ulcer in the terminal ileum. Laparoscopic ileocecal resection was performed with a preoperative diagnosis of ileal cancer. The tumor adhered to the right internal inguinal ring. We dissected the right spermatic cord involved in the tumor. The resected specimen revealed a fistula between the appendiceal orifice and ileac ulcer. Histopathological examination revealed a well differentiated tubular adenocarcinoma. We made the diagnosis of appendiceal cancer with an ileal fistula because the ileal ulcer was derived from the appendiceal site.

**Discussion:**

Most cases of appendiceal cancer with a fistula undergo laparotomy, but in selected cases, laparoscopic resection should be considered a feasible, safe, and curative procedure. Our patient underwent laparoscopic ileocecal resection, whereby the tumor and other organs with invasion were resected successfully with a negative surgical margin.

**Conclusion:**

This is the first case report of appendiceal cancer with an ileal fistula successfully treated with laparoscopic resection. Laparoscopic ileocecal resection can be applied for appendiceal cancers with a fistula by experienced surgeons with careful consideration.

## Highlights

• We experienced a case of appendiceal cancer invading the ileum with a fistula.
• This is the first case report of appendiceal cancer with an ileal fistula that successfully treated with laparoscopic resection.
• Laparoscopic resection can be a feasible, safe and curative procedure in selected cases of appendiceal cancer with a fistula.
• Laparoscopic ileocecal resection can be applied for appendiceal cancers with a fistula by experienced surgeons with careful consideration.

## 1 Introduction

Primary appendiceal cancer is an extremely rare disease, accounting for 0.1–0.8% of all appendectomies, and synchronous colon cancers are present in 10–30% of these cases [[1], [2], [3], [4]]. Appendiceal cancer constitutes approximately 0.5% of all gastrointestinal tract tumors [1,5], and most commonly develops in those 50–60 years of age [[3], [4], [5], [6]], with a slight male predominance [3,6]. There are some case reports of fistula formation in gastrointestinal tract cancers [7,8], but those for appendiceal cancer are much rarer. The most common organs forming a fistula from appendiceal cancer are the skin, colon, ileum, bladder and ovary [9]. We experienced a case of appendiceal cancer invading the ileum with a fistula who underwent successful laparoscopic ileocecal resection.

## 2 Presentation of case

**Fig. 1:**
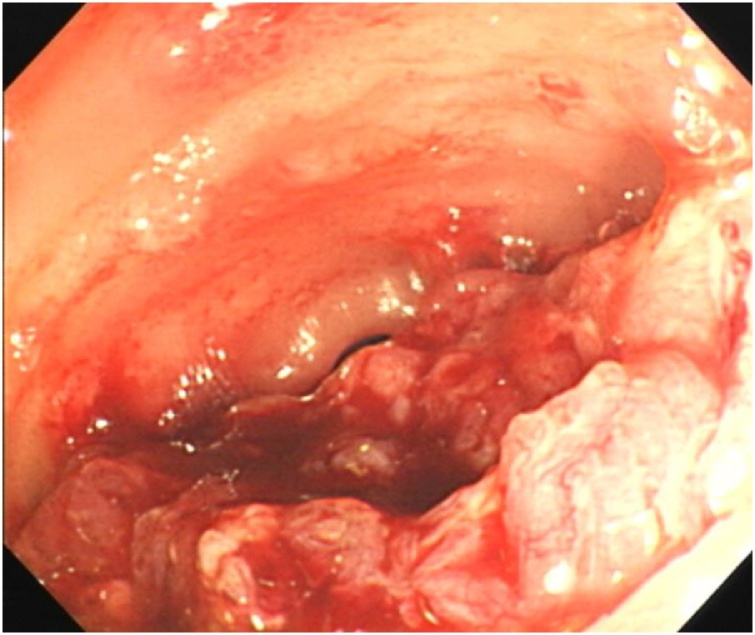
Colonoscopic view of an ulcer that oozed upon slight contact in the terminal ileum.

**Fig. 2:**
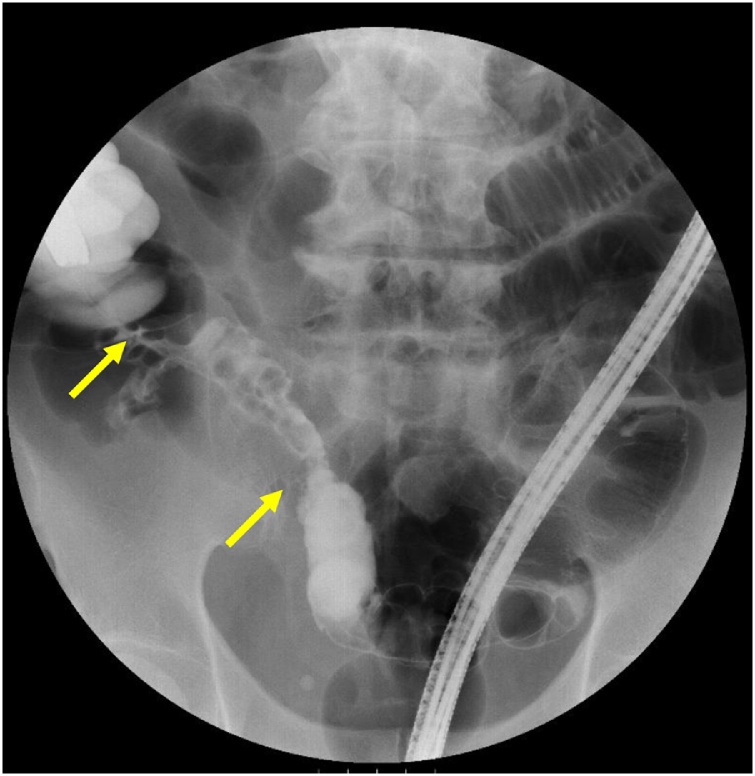
Fluoroscopic study of 5-cm narrowing at the small intestine.

**Fig. 3:**
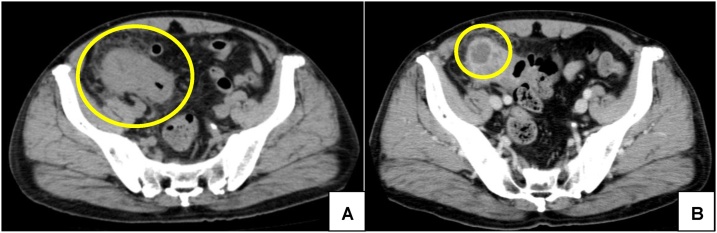
Enhanced abdominal computed tomography scan. A. 45 mm × 55 mm irregular ileocecal wall thickness with enhancement. B. Low density mass such as an abscess caudal of the tumor.

A 76-year-old man who presented with fever and abdominal pain was diagnosed with acute appendicitis and received antibiotics at a local hospital. After a few days, he received a colonoscopy, which revealed a mass in the terminal ileum, and was then referred to our hospital. He had a past medical history of hypertension and a family history of paternal pancreatic cancer and a brother with prostate cancer. Physical examination revealed a blood pressure of 140/82 mmHg, pulse rate of 82/min, and temperature of 35.5 °C. Abdominal examination revealed an elastic hard mass with mild tenderness and a diameter of about 5 cm in the right lower quadrant. His abdomen was soft and flat, and he had no peritoneal signs. Laboratory data showed a white blood cell count of 9600 μ/L, C-reactive protein value of 0.26 mg/dl and CEA level of 6.4 ng/ml (normal <5.0 ng/ml). A colonoscopy exam revealed an oozing ulcer in the terminal ileum (Fig. 1). Fluoroscopic study of the small intestine demonstrated 5-cm narrowing at the terminal ileum　(Fig. 2). Enhanced computed tomography showed 45 mm × 55 mm irregular ileocecal wall thickness with enhancement (Fig. 3). Thus, we made the preoperative diagnosis of ileal cancer.

**Fig. 4:**
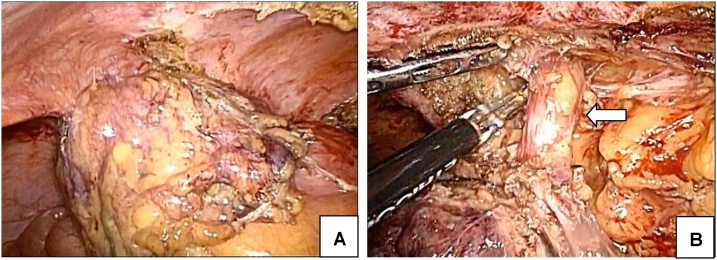
Surgical findings. A. A tumor surrounded by fatty tissue and omentum was located in the ileocecal area, and an adhesion was formed between the right internal inguinal ring. B. Right spermatic cord was dissected because it was involved in the tumor (arrow: right spermatic cord).

A laparoscopic ileocecal resection was performed after the patient gave informed consent regarding the surgical technique, possible complications, and the possibility of conversion to conventional open surgery. With the patient in a supine position and legs apart, we made an incision in the umbilicus and a 10-mm trocar was introduced. After setting the pneumoperitoneum to 10 mmHg, a 5-mm trocar was introduced intraperitoneally into the right lower abdomen and left abdomen, respectively, and a 12-mm trocar was similarly introduced into the left abdomen. A tumor surrounded by fatty tissue and omentum was located in ileocecal area and adhered to the right internal inguinal ring (Fig. 4A). We dissected the right spermatic cord because it was involved in the tumor (Fig. 4B). The external iliac artery had not been invaded. Ileocecal vessels were exposed, clipped, and dissected at the root with extensive lymph node dissection (D3). Mobilization of the right colon and division of the mesoappendix were performed with an ENSEAL (Ethicon Endo-Surgery Inc., Cincinnati, OH, USA) and a bipolar coagulation device. After complete mobilization, the umbilical incision was extended 8 cm and the Alexis O-Ring wound retractor (Applied Medical, Rancho Santa Margarita, CA, USA) was used to prevent port-site metastasis. Including the tumor, ileocecal resection with stapled functional end-to-end anastomosis was performed extracorporeally by using an Echelon Flex 60 Linear Cutter (Ethicon Endo-Surgery Inc., Cincinnati, OH, USA).

**Fig. 5:**
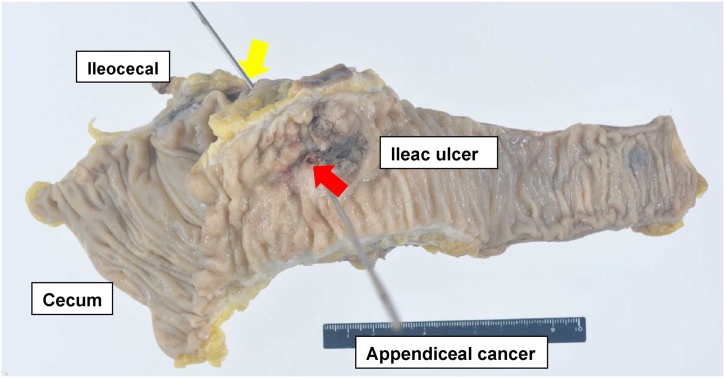
Resected specimen revealed a fistula between the appendiceal orifice and ileac ulcer. (yellow arrow, appendiceal orifice; red arrow: ileac ulcer).

**Fig. 6:**
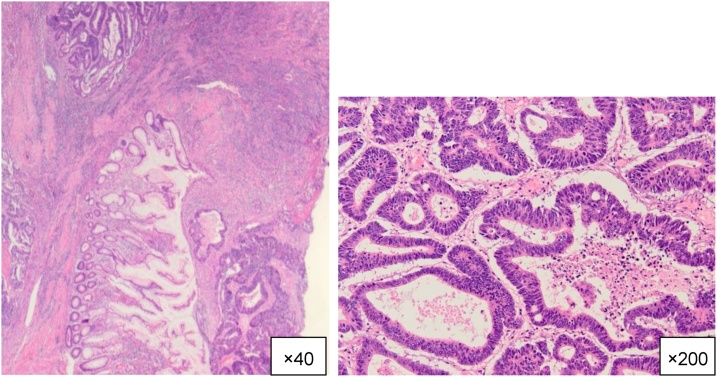
Histopathological examination revealed well differentiated tubular adenocarcinoma (hematoxylin and eosin). Tumoral tissue was revealed in the mucosal membrane of the appendix but not in the iliac ulcer. Iliac ulcer was derived from the appendiceal site.

The resected specimen revealed a fistula between the appendiceal orifice and ileac ulcer (Fig. 5). Histopathological examination showed a well differentiated tubular adenocarcinoma; tumoral tissue was revealed in the mucosal membrane of the appendix but not in the iliac ulcer (Fig. 6). Lympho-vascular invasion and lymph node metastasis were not detected. Surgical margins were free of cancer cells and no metastasis from the carcinoma was found in the lymph nodes. We diagnosed the tumor as an appendiceal cancer, T4bN0M0 Stage llC (TMN classification 7th), with a fistula of the ileum because the iliac ulcer was derived from the appendiceal site. The patient had an uneventful postoperative course, and was discharged from the hospital on the 11th day post-operation. He received adjuvant chemotherapy with XELOX (capecitabine and oxaliplatin) for 6 months. He was diagnosed with prostate cancer 10 months after surgery and received brachytherapy. Currently, he is stable and alive 42 months after surgery.

## 3 Discussion

A PubMed database search for the period from January 1980 to August 2018 was performed using the key words “appendiceal cancer” and “ileac fistula”. The search identified only one English language report which was written by Nakada et al. [10]. They reported a case of appendiceal cancer with a fistula at 2 sites in the ileum and in the cecum, transverse colon, and sigmoid colon for a total of 5 sites. A Japanese report, from 1997 by Ito et al. [9], reported 4 cases of appendiceal cancer with an ileal fistula. In these cases, only the Nakada’s case and ours, received a accurate preoperative diagnosis of appendiceal cancer.

Appendiceal cancer most often presents with acute appendicitis or a palpable abdominal mass, and some of these tumors are entirely asymptomatic. Preoperative diagnosis is difficult because of poorly defined characteristic findings. Wang et al. [11] reported the feasibility of using CT to differentiate malignant from benign lesions in patients with appendiceal mucoceles. They suggested that irregular walls and soft-tissue thickening were most likely to be associated with malignancy (p < 0.005). In another paper, 75 primary neoplasms of the appendix were analyzed, and it was reported that a larger size was a useful diagnostic indicator [12]. Our case had a large area of irregular ileocecal wall thickness with enhancement on enhanced CT. Recently, the benefits of diagnostic laparoscopic surgery was reported [13].

Surgical resection is the only curative treatment for appendiceal cancer. In previous reports, all cases received surgery, with 4 of the 5 reported patients undergoing open radical surgery. One received non-curative open surgery with ileocecal resection and closing of other sites of the fistula. Our case underwent laparoscopic ileocecal resection, whereby the tumor and other organs with invasion were resected successfully with a negative surgical margin. Most cases with fistula undergo laparotomic surgery, however, in selected cases, laparoscopic resection can also be feasible. Indeed, some surgeons have reported the beneficial aspects of laparoscopic resection of appendiceal tumors [[14], [15], [16], [17]]. The laparoscopic approach can provide good surgical information about the peritoneal cavity without requiring a large incision, it can be less invasive with comparable oncologic outcomes and earlier recovery [15]. The laparoscopic view provides adequate surgical information in evaluating the tumor burden and margins macroscopically, which leads to en bloc surgical resection of the tumor. In cases with an invasive margin, surgeons should resect the intestine sufficiently away from the lesion, that is, by at least 10 cm. It is sometimes difficult to discriminate hard inflammatory tissue from a cancerous lesion, even in such a case, laparoscopy is feasible because endo-laparoscopic forceps can provide surgeons with sufficient tactile information. Furthermore, a laparoscopic approach provides a magnified view to prevent inadvertent damage to other organs such as the ureter and has advantages in the delamination of the tumor and retroperitoneum. We previously experienced a case of appendicitis with psoas abscess that was successfully treated by laparoscopic surgery that enable to detect the proper resection area [18].

Regarding oncologic safety, extreme attention must be paid not to disseminate malignant cell intraperitoneally. Surgeons should use non-traumatic endo-laparoscopic forceps in order not to crush the tumor. A wound protector or a plastic pouch should be used to transport specimens. Surgeons should discuss carefully with surgical colleagues whether to convert to open surgery in cases that tumor adhesion or extension of the intestine prevents a good surgical view. When complying with these suggestions, the laparoscopic approach not only allows for resection of early appendiceal cancer but also for advanced disease, such as with a fistula with other organs. Appendiceal cancer tends to be associated with a poor prognosis, and fistula formation usually indicates deep invasion and advanced cancer. However, this may not always mean that non-curative surgery can be undertaken, because fistula formation sometimes prevents peritoneal dissemination. A previously reported patient has been alive for 9 years after surgery without recurrence [12].

## 4 Conclusion

This is the first case report of appendiceal cancer with an ileal fistula successfully treated with laparoscopic resection. Our patient underwent laparoscopic ileocecal resection, whereby the tumor and other organs with invasion were resected successfully with a negative surgical margin. In selected cases, laparoscopic ileocecal resection can be applied for appendiceal cancers with a fistula by experienced surgeons with careful consideration.

## Conflicts of interest

We have no conflicts of interest to disclose related to this work.

## Sources of funding

We have no funding for this work.

## Ethical approval

Ethical approval is not needed for this case report as patient consent.

## Consent

Written informed consent was obtained from the patient for publication of this case report and accompanying images. A copy of the written consent is available for review by the Editor-in-Chief of this journal on request.

## Author contribution

JM and YS drafted the manuscript. KK, YS, HH, MS, SK, YM, KY, TN, SS and YK contributed to patient care. JM, YS and KK performed the literature search. YS, KK and YK participated in the critical revision of the manuscript. All authors have read and approved the final manuscript.

## Registration of research studies

It is the institutional policy at Kobe University that a single case report (three or fewer cases) does not require an Institutional Review Board review or a Research Registry Number. This is also in compliance with the Japanese Care Act and the provisions outlined by Japanese Medical Association at the International Conference at JMA, Japan 2017. (https://www.med.or.jp/english/wma_pdf.pdf).

The patient in the case report has been completely de-identified, no information or photos in the report can be used to trace the patient's identity and the report is not an investigation of an FDA regulated product, and therefore is considered as an exempt study/report. All relevant privacy regulations, including the HIPAA privacy policy have been followed in the preparation of this report.

## Guarantor

I, Junko Mukohyama, MD, PhD, am affiliated with Kobe University and in full compliance with all Rules and Regulations governing patient care. I accept full responsibility for the work and/or the conduct of the study, had access to the data and controlled the decision to publish.

## Provenance and peer review

Not commissioned, externally peer reviewed.
